# Supplementary material for: Going New Places: Successful Adaptation and Genomic Integrity of Grain Amaranth in India
Source: Evol Appl. 2025 Jun 27;18(7):e70124. doi: 10.1111/eva.70124 (PMC12204849; doi:10.1111/eva.70124)
Supplement: Supplementary file 1 — Data S1. [file EVA-18-e70124-s003.pdf]

Supplement

Supplementary Figures

A

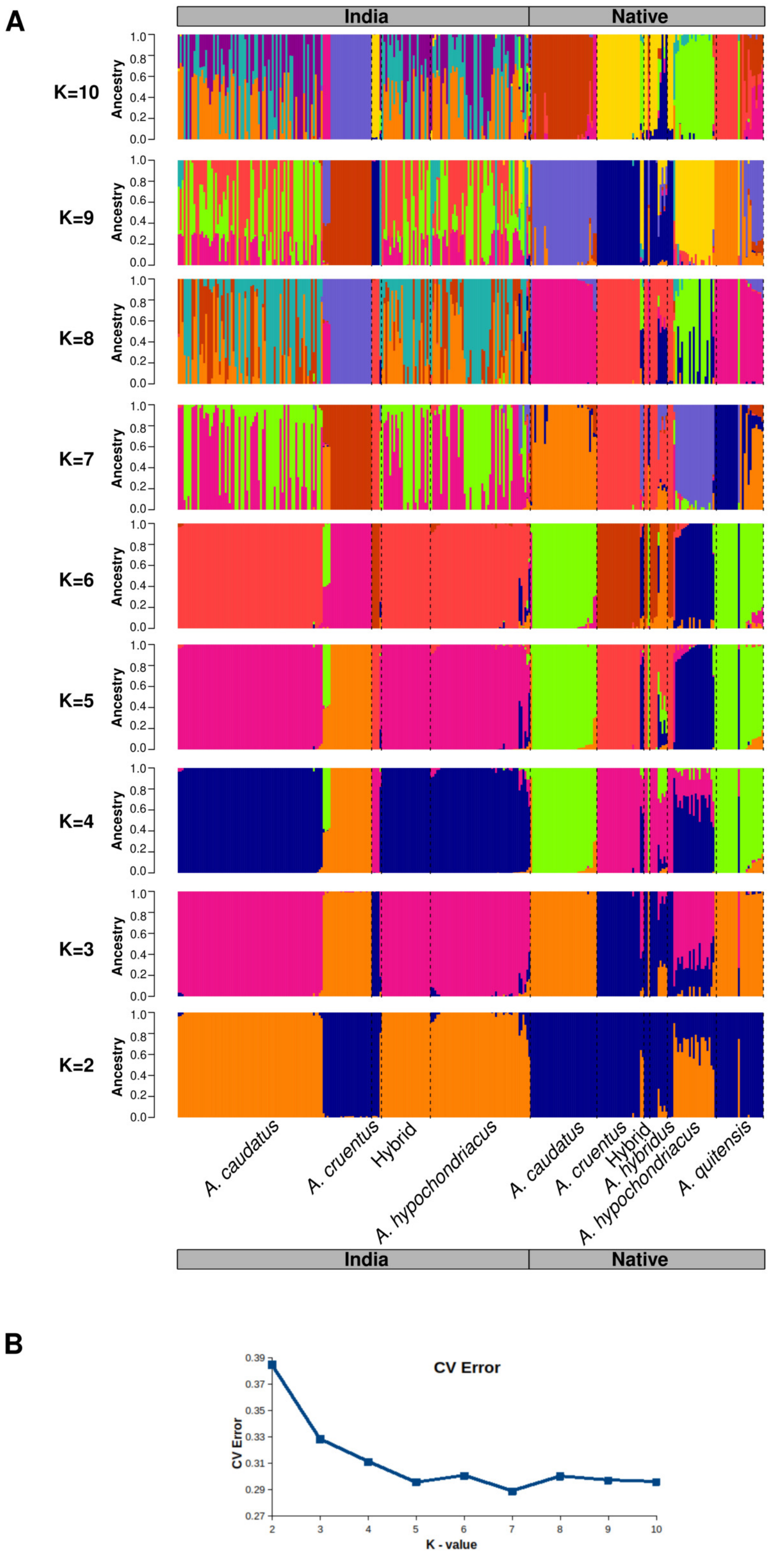

Figure S1 Population structure of grain amaranth (A) Bar-plot representation of individual ancestry proportion of Indian and Native grain amaranth accessions estimated with ADMIXTURE for different K. (B) Scatter plot representation of cross-validation (CV) error at different K values.

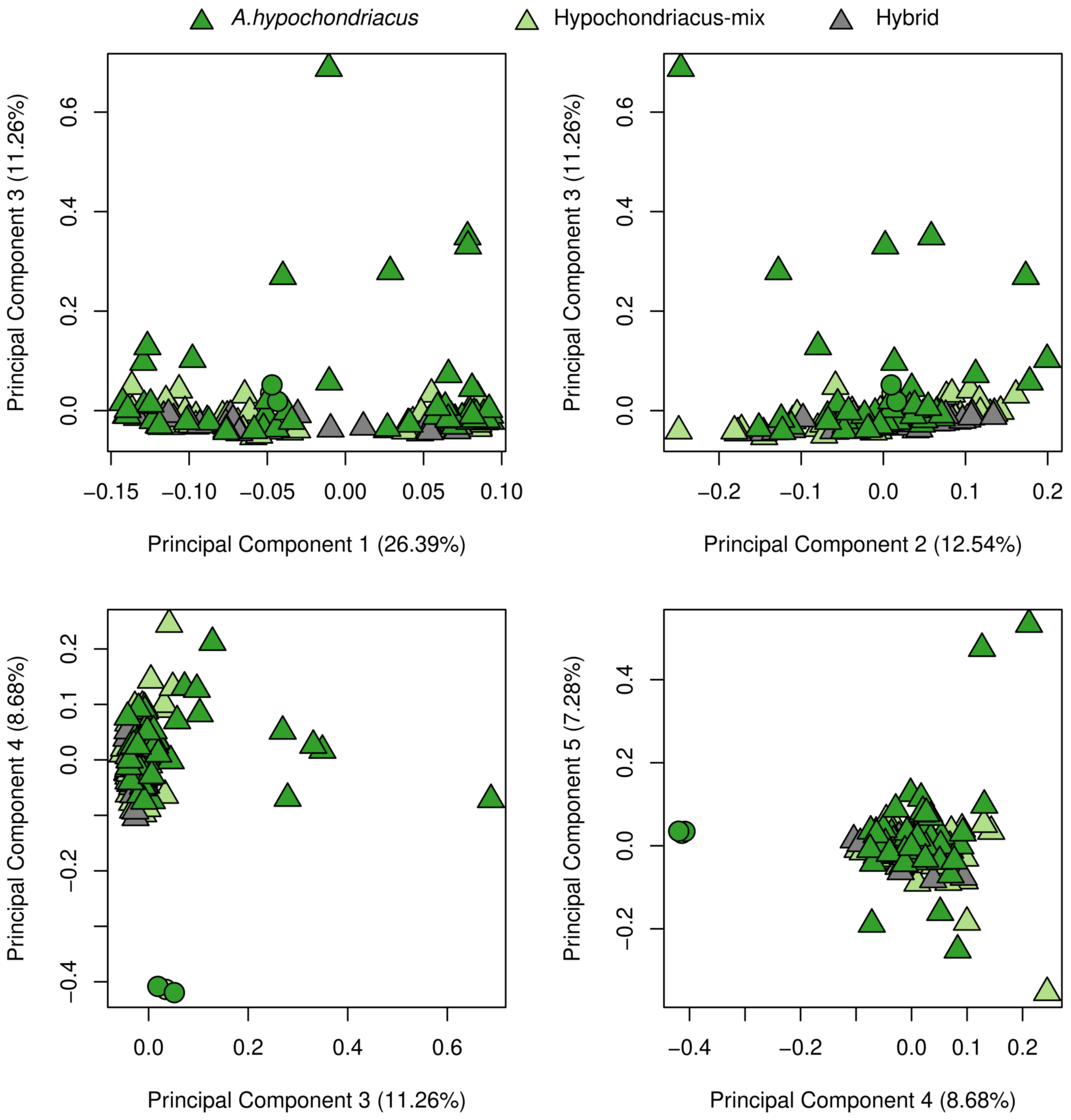

Figure S2 PCA biplot with only *A. hypochondriacus*, Hypochondriacus-mix and Hybrid accessions. Lack of clustering pattern supports the lack of population differentiation

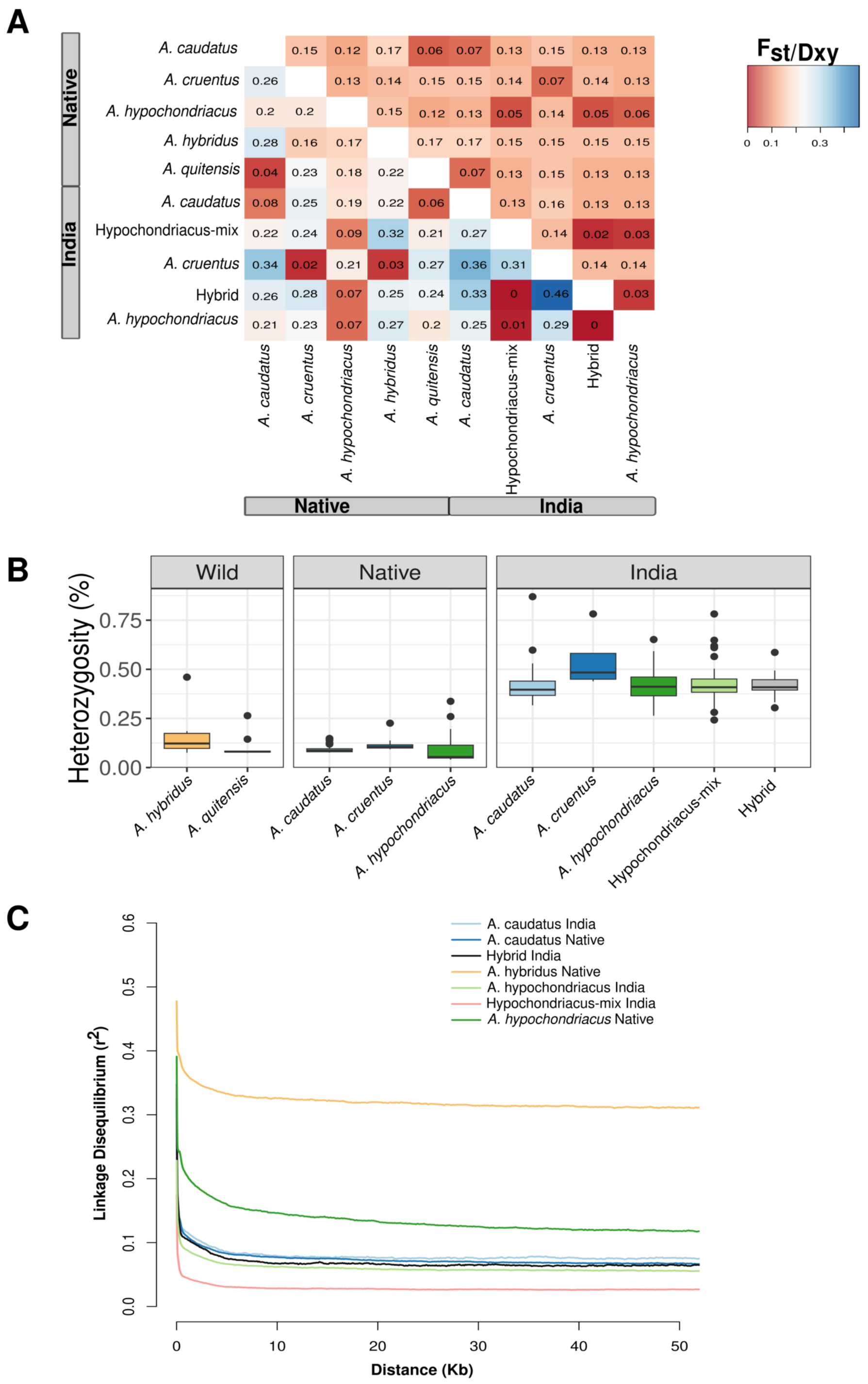

Figure S3 Genetic diversity parameters (A) Heatmap representing the Fst and Dxy statistic between each pair of population from native and India. The average values of Fst and Dxy are shown in the lower and upper matrix, respectively. (B) Estimate of global observed heterozygosity (in percentage) for all the populations (C) Plot representing linkage disequilibrium decay for all the populations.

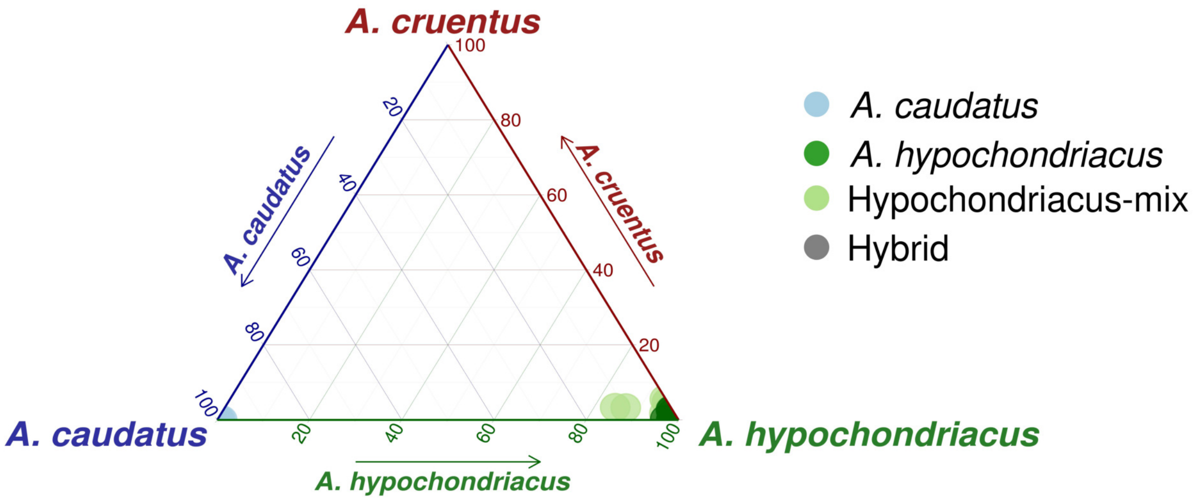

Figure S4 Ternary plot representation for ELAI results to depict the ancestry proportion for Indian accessions using native species group as source.Each corner represent each of the three domesticate species. Concentration at the edges represent no admixture.

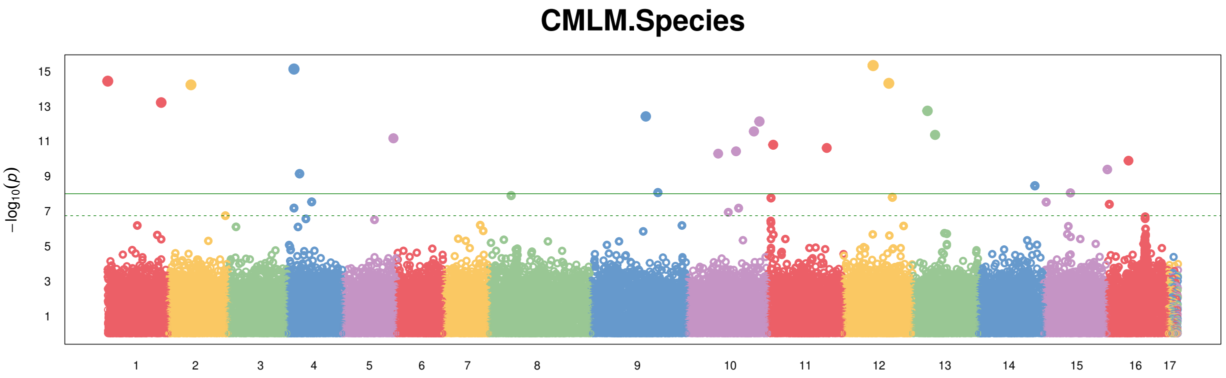

Figure S5 Manhattan plot for genome-wide association mapping with taxonomic species characterization as phenotype. Only accessions of *A. caudatus* and *A. hypochondriacus* form the two ranges (Native and India) were used. The horizontal solid line indicates a significance level of 1E-08. Each dot is a SNP and dots above the line represent significantly associated SNPs.

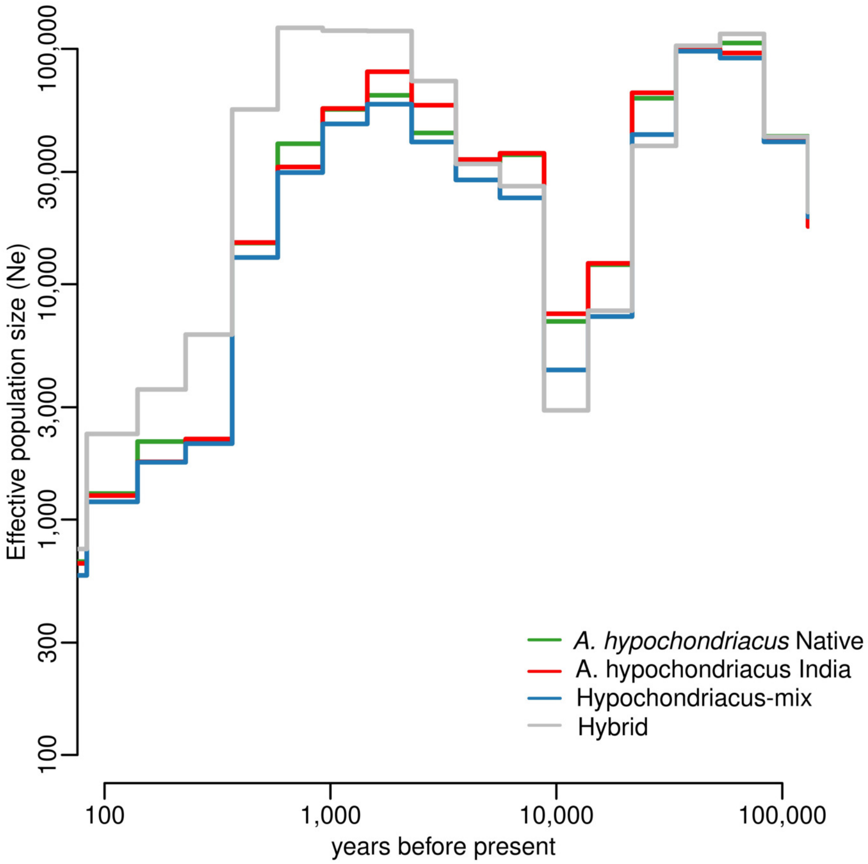

Figure S6 Step-plot representation of effective population size estimated using popsizeABC. Generation time was considered as one generation per year. Reduction in effective population size represents protracted bottlenecks.

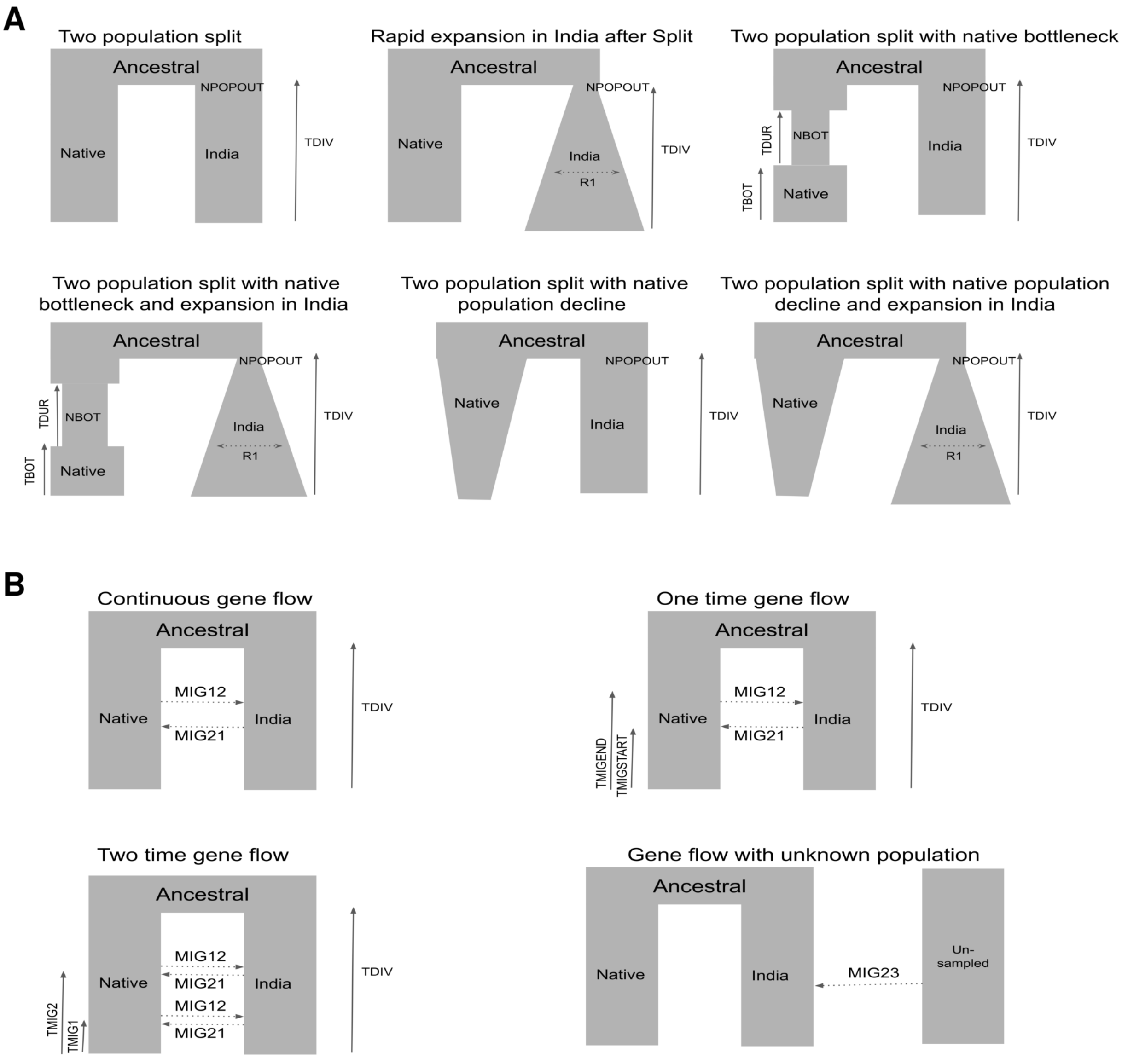

Figure S7 Demographic models tested using fastsimcoal2 (A) Six base models, (B) Models with three gene-flow scenarios. Ancestral = Effective population size of ancestral population before split; Native = Effective population size of native population; India = Effective population size of Indian population; NPOPOUT = Introduced population size; Tdiv = Time of split/divergence; R1 = rate of population expansion; NBOT = size of bottleneck; TBOT = time of bottleneck start; TDUR = Duration of bottleneck; MIG12 = migration rate from native to India; MIG21 = migration rate from India to native; MIG23 = migration rate from unknown population; TSTART = time of start of migration; TEND = time of end of migration; TSTART2 = time of start of second migration; TEND2 = time of end of second migration event.

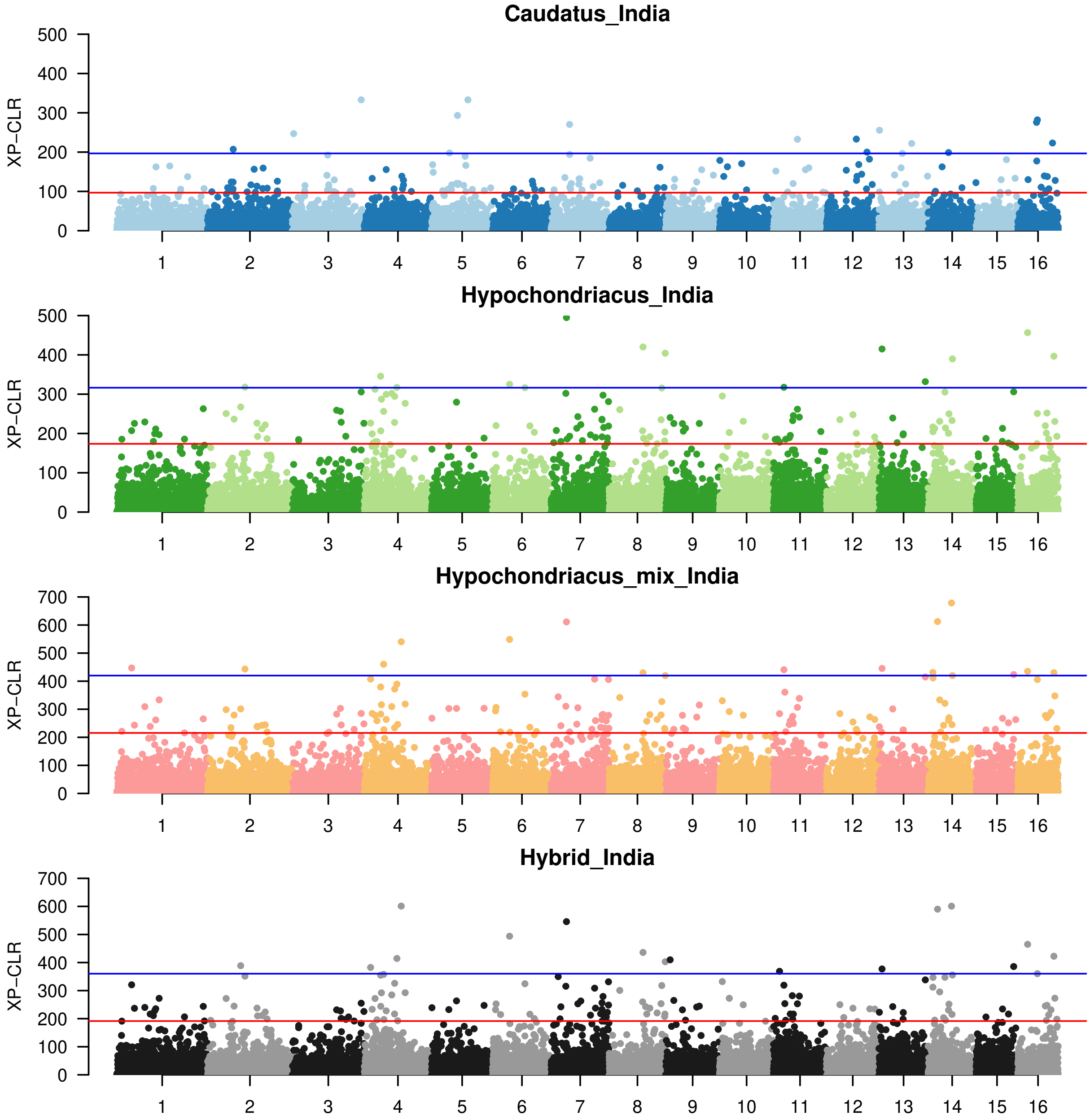

Figure S8 Manhattan plot representation of genome-wide distribution of XP-CLR statistic for two species using their respective native counter-part as reference population. For Hypochondriacus-mix and hybrid, *A. hypochondriacus* form native was taken as reference. Red line represent top 1 percentile and blue line represents 0.1 percentile.

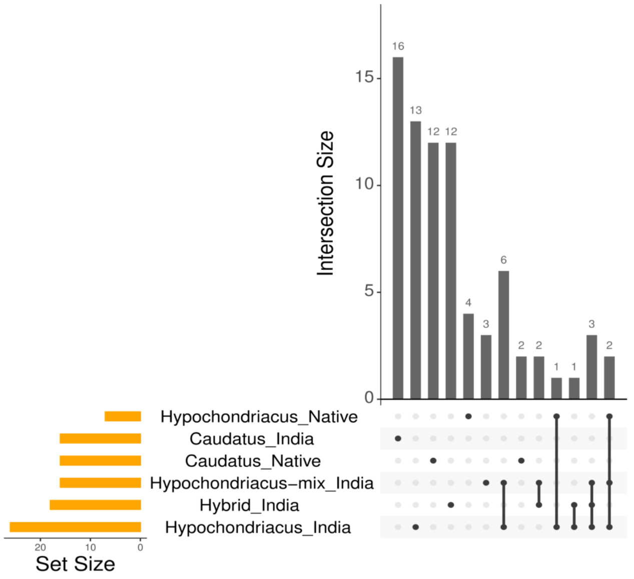

Figure S9 Shared putative selective sweep regions identified as top 1 percentile from genome-wide XP-CLR outlier with those identified per population (population specific) by RAiSD

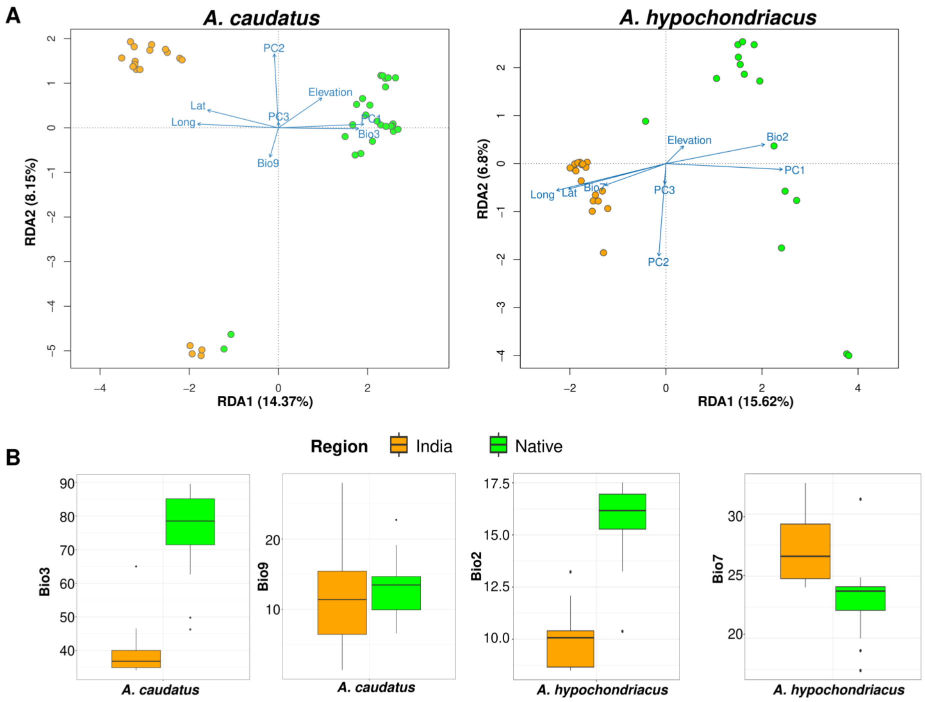

Figure S10 Climate-Genotype association (A) RDA bi-plot representing association of accessions (samples) with the two significantly associated bioclimatic variables for the two species, (B) Boxplot representation of the significantly associated bioclimatic variables in two regions (Native and India) for the two species (Bio2 = Mean diurnal temperature range, Bio7 = Temperature annual range, Bio3 = Isothermality and Bio9 = Mean temperature of the driest quarter).

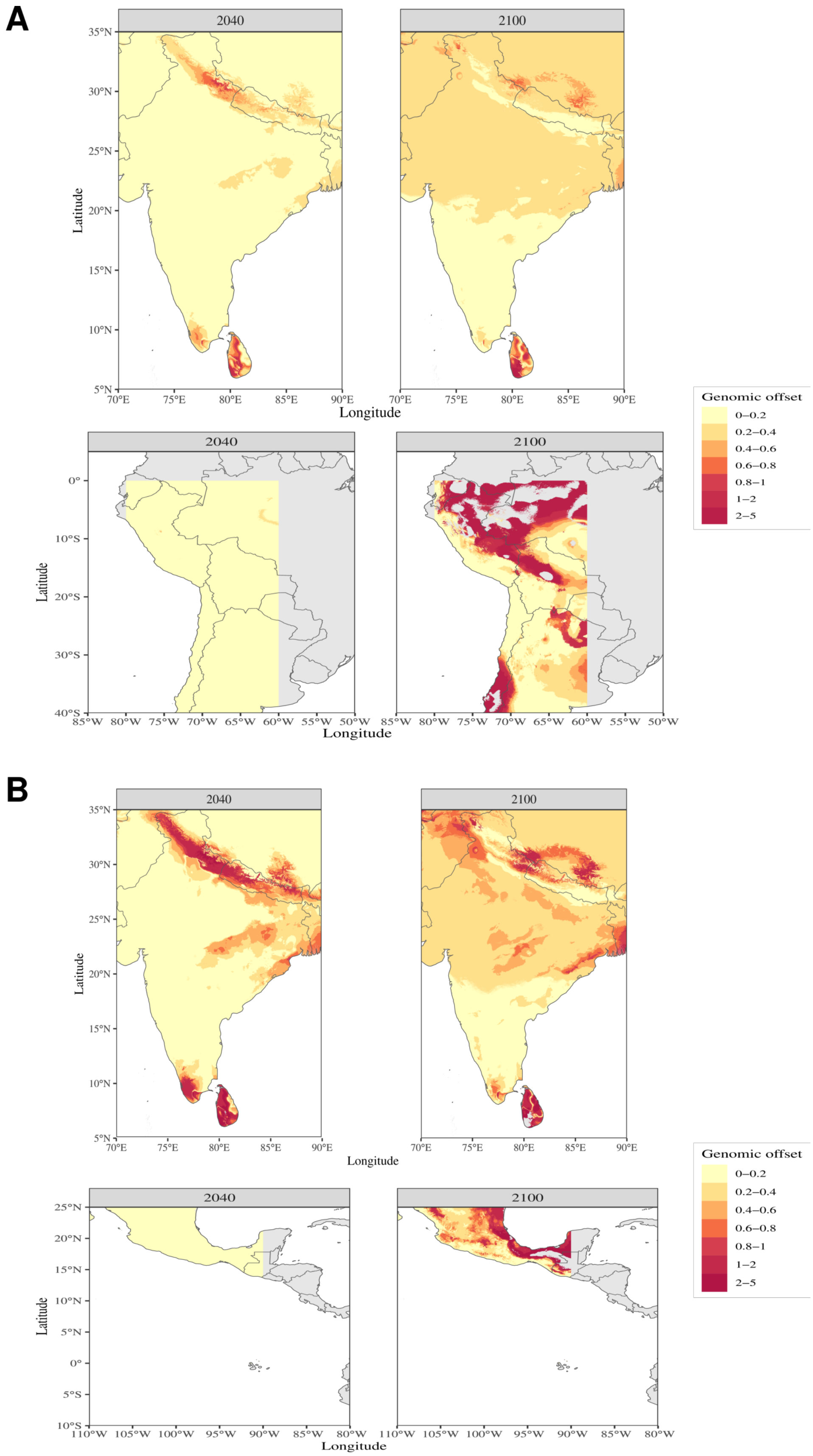

Figure S11 Genetic offset predicted using adaptive index calculated from associated climatic variable and GEA loci in the native and introduced range for two species. (A) For *A. caudatus*, top panel shows predictions for the introduced range (India), while the lower panel represents the native range (South America) and (B) For *A. hypochondriacus* top panel depicts the introduced range (India), and the lower panel shows the native range (Central America). Genomic offset was calculated for two future scenarios of 2040 and 2100. Darker color represents higher offset.

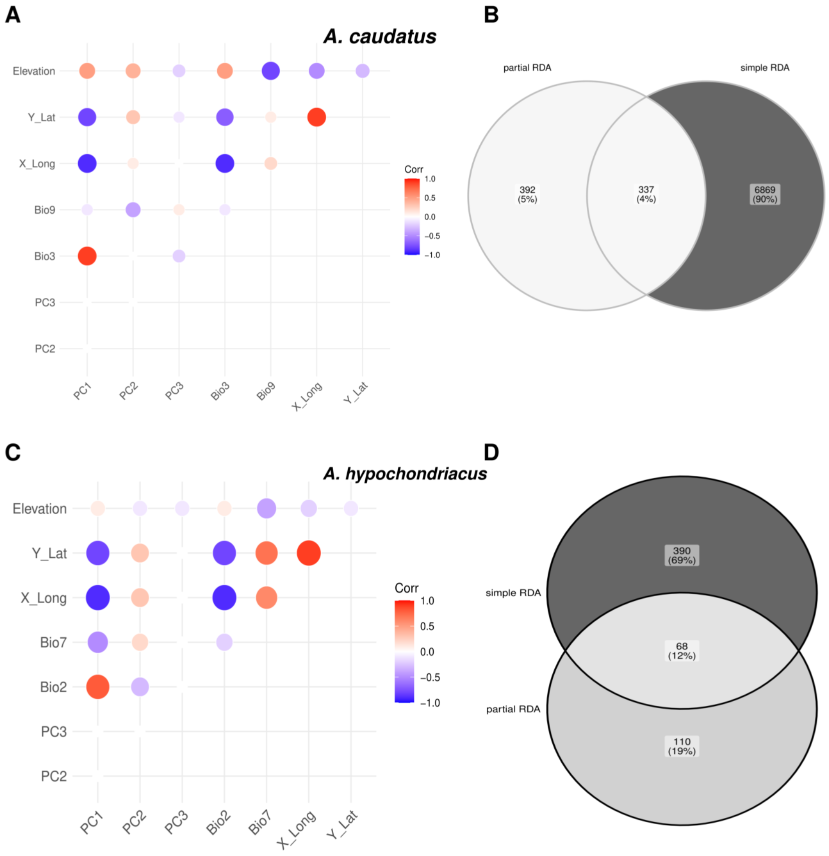

Figure S12 Correlation among the predictor variables of RDA (A and C) and overlap between constrained and unconstrained RDA analysis (B and D) for the two species: (A-B) *A. caudatus* and (C-D) *A. hypochondriacus*.

Supplementary Tables

Table S1 List of accessions used in the study along with new assigned cluster and mapping summary.

USDA Accession No.	Sample Name	Species (USDA)	Country Origin	Region	Mean cov.	% Aligned	Fraction SNPs missing	Included in study	New assigned cluster
PI 274278	AM_00546	hypochondriacus	IND	India	8.7X	98.5%	9.1	Yes	hypochondriacus_India
PI 480516	AM_00547	caudatus	IND	India	7.6X	98.5%	13.3	Yes	hypochondriacus-mix_India
PI 480557	AM_00548	hypochondriacus	IND	India	9.5X	98.8%	7.3	Yes	hypochondriacus_India
PI 480569	AM_00549	hypochondriacus	IND	India	7.1X	98.9%	10.7	Yes	hypochondriacus_India
PI 481091	AM_00550	hypochondriacus	IND	India	10.3X	98.8%	6.9	Yes	hypochondriacus_India
PI 481184	AM_00551	hypochondriacus	IND	India	8.4X	98.6%	11.1	Yes	hypochondriacus_India
PI 481336	AM_00552	hypochondriacus	IND	India	6.7X	98.6%	17.2	Yes	hypochondriacus_India
PI 669884	AM_00553	hypochondriacus	IND	India	8.8X	98.4%	11	Yes	hypochondriacus_India
PI 669931	AM_00554	hypochondriacus	IND	India	4.6X	98.6%	27.2	Yes	hypochondriacus_India
PI 566897	AM_00558	cruentus	IND	India	7.6X	98.1%	18.3	Yes	cruentus_India
Ames 15204	AM_00601	hypochondriacus	IND	India	4.1X	99.1%	25.2	Yes	hypochondriacus_India
Ames 15210	AM_00602	hypochondriacus	IND	India	1.1X	99.3%	64.3	Yes	hypochondriacus_India
Ames 15301	AM_00604	hypochondriacus	IND	India	9.8X	98.4%	8.2	Yes	hypochondriacus_India
Ames 2257	AM_00607	hypochondriacus	IND	India	6.2X	98.6%	16.7	Yes	hypochondriacus_India
Ames 5138	AM_00609	hypochondriacus	IND	India	10.0X	98.7%	7.4	Yes	hypochondriacus_India
Ames 5145	AM_00610	hypochondriacus	IND	India	8.8X	98.7%	9.3	Yes	hypochondriacus_India
Ames 5386	AM_00613	cruentus	IND	India	8.5X	98.1%	19	Yes	cruentus_India
Ames 5585	AM_00637	hypochondriacus	IND	India	10.0X	98.4%	7.8	Yes	hypochondriacus_India
Ames 5586	AM_00638	hypochondriacus	IND	India	10.2X	98.4%	8.5	Yes	hypochondriacus_India
Ames 5587	AM_00639	hypochondriacus	IND	India	7.4X	98.4%	13.8	Yes	hypochondriacus_India
Ames 5588	AM_00640	hypochondriacus	IND	India	9.1X	98.4%	7.9	Yes	hypochondriacus_India
Ames 5593	AM_00643	hypochondriacus	IND	India	10.7X	98.6%	6.1	Yes	hypochondriacus_India
Ames 5603	AM_00644	hypochondriacus	IND	India	5.1X	99.2%	18.3	Yes	hypochondriacus_India
Ames 5608	AM_00645	hypochondriacus	IND	India	9.8X	98.6%	7.1	Yes	hypochondriacus_India
Ames 5611	AM_00646	hypochondriacus	IND	India	8.1X	98.3%	12.4	Yes	hypochondriacus_India
Ames 5612	AM_00647	hypochondriacus	IND	India	10.0X	98.6%	7.6	Yes	hypochondriacus_India
Ames 5613	AM_00648	hypochondriacus	IND	India	3.9X	98.9%	31.2	Yes	hypochondriacus_India
PI 166045	AM_00664	caudatus	IND	India	7.9X	98.3%	19.6	Yes	caudatus_India
PI 166107	AM_00665	caudatus	IND	India	8.3X	98.0%	20.8	Yes	caudatus_India
PI 175039	AM_00666	caudatus	IND	India	9.4X	98.2%	17.2	Yes	caudatus_India
PI 175040	AM_00667	hypochondriacus	IND	India	7.5X	98.7%	16.7	Yes	hypochondriacus_India
PI 274277	AM_00668	hypochondriacus	IND	India	9.6X	98.3%	8.7	Yes	hypochondriacus_India
PI 274278	AM_00669	hypochondriacus	IND	India	6.6X	98.5%	16.3	Yes	hypochondriacus_India
PI 274279	AM_00670	hypochondriacus	IND	India	8.0X	97.9%	12.1	Yes	hypochondriacus_India
PI 288278	AM_00671	cruentus	IND	India	7.8X	98.4%	18.9	Yes	cruentus_India

PI 288279	AM_00672	caudatus	IND	India	8.7X	98.2%	18	Yes	caudatus_India
PI 480487	AM_00675	caudatus	IND	India	8.4X	98.6%	9.6	Yes	hypochondriacus-mix_India
PI 480491	AM_00676	caudatus	IND	India	11.1X	99.0%	4.7	Yes	hypochondriacus-mix_India
PI 480493	AM_00677	caudatus	IND	India	0.9X	98.9%	59.6	Yes	hypochondriacus-mix_India
PI 480494	AM_00678	caudatus	IND	India	7.4X	98.6%	12.3	Yes	hypochondriacus-mix_India
PI 480498	AM_00679	caudatus	IND	India	8.3X	98.6%	10.3	Yes	hypochondriacus-mix_India
PI 480500	AM_00680	caudatus	IND	India	6.7X	98.7%	14.4	Yes	hypochondriacus-mix_India
PI 480503	AM_00681	caudatus	IND	India	8.3X	98.7%	15.4	Yes	hypochondriacus-mix_India
PI 480514	AM_00682	caudatus	IND	India	10.5X	98.4%	13.9	Yes	caudatus_India
PI 480516	AM_00683	caudatus	IND	India	8.1X	99.1%	8.4	Yes	hypochondriacus-mix_India
PI 480525	AM_00684	caudatus	IND	India	6.0X	99.0%	17.9	Yes	hypochondriacus-mix_India
PI 480546	AM_00685	caudatus	IND	India	8.6X	98.4%	19.4	Yes	caudatus_India
PI 480568	AM_00686	caudatus	IND	India	7.2X	98.6%	14	Yes	hypochondriacus-mix_India
PI 480574	AM_00687	caudatus	IND	India	9.6X	99.1%	6.3	Yes	hypochondriacus-mix_India
PI 480575	AM_00688	caudatus	IND	India	4.2X	98.9%	30.6	Yes	hypochondriacus-mix_India
PI 480576	AM_00689	caudatus	IND	India	1.0X	98.6%	63.6	Yes	caudatus_India
PI 480577	AM_00690	caudatus	IND	India	7.0X	98.6%	15.7	Yes	hypochondriacus-mix_India
PI 480591	AM_00691	caudatus	IND	India	5.9X	98.8%	17.1	Yes	hypochondriacus-mix_India
PI 480596	AM_00692	caudatus	IND	India	10.0X	98.6%	5.3	Yes	hypochondriacus-mix_India
PI 480616	AM_00693	caudatus	IND	India	7.3X	98.2%	19	Yes	caudatus_India
PI 480623	AM_00694	caudatus	IND	India	7.8X	98.8%	12.1	Yes	hypochondriacus-mix_India
PI 480655	AM_00695	caudatus	IND	India	8.9X	99.0%	6.7	Yes	hypochondriacus-mix_India
PI 480656	AM_00696	caudatus	IND	India	1.6X	96.9%	42.7	Yes	hypochondriacus-mix_India
PI 480659	AM_00697	caudatus	IND	India	7.2X	98.9%	10.1	Yes	hypochondriacus-mix_India
PI 480660	AM_00698	caudatus	IND	India	7.6X	98.9%	10.8	Yes	hypochondriacus-mix_India
PI 480666	AM_00699	caudatus	IND	India	1.6X	96.8%	41.4	Yes	hypochondriacus-mix_India
PI 480674	AM_00700	caudatus	IND	India	5.6X	98.6%	17.7	Yes	hypochondriacus-mix_India

PI 480675	AM_00701	caudatus	IND	India	1.8X	98.6%	38.7	Yes	hypochondriacus-mix_India
PI 480676	AM_00702	caudatus	IND	India	10.1X	98.5%	5.6	Yes	hypochondriacus-mix_India
PI 480677	AM_00703	caudatus	IND	India	7.8X	98.5%	9.2	Yes	hypochondriacus-mix_India
PI 480679	AM_00704	hybr.	IND	India	6.9X	99.0%	9.8	Yes	hybrid_India
PI 480742	AM_00705	caudatus	IND	India	7.5X	98.6%	11.9	Yes	hypochondriacus-mix_India
PI 480748	AM_00706	caudatus	IND	India	1.6X	99.2%	43.5	Yes	hypochondriacus-mix_India
PI 480749	AM_00707	caudatus	IND	India	0.8X	93.3%	64.6	Yes	hypochondriacus-mix_India
PI 480750	AM_00708	caudatus	IND	India	2.4X	99.3%	39.4	Yes	hypochondriacus-mix_India
PI 480760	AM_00709	caudatus	IND	India	8.3X	98.9%	7.5	Yes	hypochondriacus-mix_India
PI 480762	AM_00710	hybr.	IND	India	5.9X	98.7%	19.4	Yes	hybrid_India
PI 480767	AM_00711	hybr.	IND	India	6.8X	98.6%	14.9	Yes	hybrid_India
PI 480788	AM_00712	caudatus	IND	India	1.5X	99.0%	44.4	Yes	hypochondriacus-mix_India
PI 480797	AM_00713	hybr.	IND	India	8.6X	98.7%	8	Yes	hybrid_India
PI 480804	AM_00714	caudatus	IND	India	0.9X	97.1%	64	Yes	hypochondriacus-mix_India
PI 480805	AM_00715	caudatus	IND	India	8.6X	98.9%	7	Yes	hypochondriacus-mix_India
PI 480816	AM_00716	caudatus	IND	India	1.6X	99.1%	43.1	Yes	hypochondriacus-mix_India
PI 480820	AM_00717	hybr.	IND	India	3.8X	99.4%	25.2	Yes	hybrid_India
PI 480822	AM_00718	caudatus	IND	India	0.9X	99.4%	63.1	Yes	hypochondriacus-mix_India
PI 480826	AM_00719	hybr.	IND	India	8.6X	99.0%	6.5	Yes	hybrid_India
PI 480827	AM_00720	hybr.	IND	India	5.0X	98.6%	16.5	Yes	hybrid_India
PI 480828	AM_00721	caudatus	IND	India	5.1X	98.3%	20.4	Yes	hypochondriacus-mix_India
PI 480833	AM_00722	caudatus	IND	India	6.3X	98.4%	15.6	Yes	hypochondriacus-mix_India
PI 480834	AM_00723	hybr.	IND	India	9.4X	98.5%	6.5	Yes	hybrid_India
PI 480836	AM_00724	caudatus	IND	India	8.8X	99.0%	7.1	Yes	hypochondriacus-mix_India
PI 480837	AM_00725	hybr.	IND	India	6.4X	98.5%	14.7	Yes	hybrid_India
PI 480845	AM_00726	hybr.	IND	India	4.9X	98.7%	17	Yes	hybrid_India
PI 480848	AM_00727	caudatus	IND	India	2.1X	99.1%	33.9	Yes	hypochondriacus-mix_India
PI 480851	AM_00728	hybr.	IND	India	10.5X	99.0%	5	Yes	hybrid_India
PI 480854	AM_00729	caudatus	IND	India	8.4X	98.6%	7.6	Yes	hypochondriacus-mix_India

PI 480855	AM_00730	caudatus	IND	India	2.2X	98.6%	31.7	Yes	hypochondriacus-mix_India
PI 480861	AM_00732	hybr.	IND	India	1.0X	98.8%	56.8	Yes	hybrid_India
PI 480864	AM_00733	caudatus	IND	India	0.3X	98.9%	84.6	No	NA
PI 480902	AM_00734	hybr.	IND	India	1.0X	98.9%	58.1	Yes	hybrid_India
PI 480923	AM_00735	hybr.	IND	India	1.3X	98.7%	47.9	Yes	hybrid_India
PI 480993	AM_00736	caudatus	IND	India	0.5X	99.0%	75.9	Yes	hypochondriacus-mix_India
PI 480994	AM_00737	caudatus	IND	India	7.6X	98.8%	8.3	Yes	hypochondriacus-mix_India
PI 480999	AM_00738	caudatus	IND	India	7.5X	99.1%	9.4	Yes	hypochondriacus-mix_India
PI 481000	AM_00739	caudatus	IND	India	0.4X	98.9%	78.4	Yes	hypochondriacus-mix_India
PI 481002	AM_00740	hybr.	IND	India	0.8X	98.6%	62.6	Yes	hybrid_India
PI 481004	AM_00741	caudatus	IND	India	0.9X	88.9%	58.1	Yes	hypochondriacus-mix_India
PI 481005	AM_00742	hybr.	IND	India	0.5X	98.9%	76.9	Yes	hybrid_India
PI 481006	AM_00743	caudatus	IND	India	0.5X	99.1%	74.5	Yes	hypochondriacus-mix_India
PI 481007	AM_00744	caudatus	IND	India	1.0X	99.0%	55.5	Yes	hypochondriacus-mix_India
PI 481012	AM_00745	hybr.	IND	India	0.2X	98.5%	88.7	No	NA
PI 481019	AM_00746	hybr.	IND	India	9.2X	98.8%	8.4	Yes	hybrid_India
PI 481020	AM_00747	caudatus	IND	India	1.5X	98.7%	47.8	Yes	hypochondriacus-mix_India
PI 481022	AM_00748	hybr.	IND	India	1.7X	98.9%	41.3	Yes	hybrid_India
PI 481024	AM_00749	hybr.	IND	India	1.3X	98.8%	48.5	Yes	hybrid_India
PI 481025	AM_00750	caudatus	IND	India	0.6X	99.0%	71.6	Yes	hypochondriacus-mix_India
PI 481026	AM_00751	caudatus	IND	India	0.8X	99.0%	62.6	Yes	hypochondriacus-mix_India
PI 481028	AM_00752	caudatus	IND	India	0.3X	98.8%	83.4	No	NA
PI 481030	AM_00753	hybr.	IND	India	1.7X	98.0%	42.4	Yes	hybrid_India
PI 481032	AM_00754	caudatus	IND	India	8.5X	98.5%	7.6	Yes	hypochondriacus-mix_India
PI 481033	AM_00755	hybr.	IND	India	0.6X	98.9%	71.3	Yes	hybrid_India
PI 481037	AM_00756	caudatus	IND	India	0.5X	98.4%	77.3	Yes	caudatus_India
PI 481040	AM_00757	caudatus	IND	India	1.4X	98.3%	44.4	Yes	hypochondriacus-mix_India
PI 481041	AM_00758	caudatus	IND	India	8.2X	98.8%	13.1	Yes	hypochondriacus-mix_India
PI 481042	AM_00759	hypochondriacus	IND	India	1.9X	99.2%	36	Yes	hypochondriacus_India
PI 481043	AM_00760	caudatus	IND	India	0.6X	98.6%	72.3	Yes	hypochondriacus-mix_India
PI 481045	AM_00762	caudatus	IND	India	1.0X	99.1%	58.6	Yes	hypochondriacus-mix_India

PI 481046	AM_00763	caudatus	IND	India	0.7X	98.2%	70.5	Yes	caudatus_India
PI 481047	AM_00764	caudatus	IND	India	4.0X	98.7%	25.1	Yes	caudatus_India
PI 481048	AM_00765	caudatus	IND	India	8.3X	98.2%	12.4	Yes	hypochondriacus-mix_India
PI 481051	AM_00766	caudatus	IND	India	0.3X	98.1%	86.8	No	NA
PI 481052	AM_00767	caudatus	IND	India	2.7X	98.7%	25.5	Yes	hypochondriacus-mix_India
PI 481053	AM_00768	caudatus	IND	India	0.9X	99.1%	58.9	Yes	hypochondriacus-mix_India
PI 481054	AM_00769	caudatus	IND	India	0.7X	98.7%	68.3	Yes	hypochondriacus-mix_India
PI 481056	AM_00770	hybr.	IND	India	0.8X	98.8%	64.8	Yes	hybrid_India
PI 481057	AM_00771	caudatus	IND	India	0.4X	98.2%	82	No	NA
PI 481058	AM_00772	caudatus	IND	India	5.3X	98.7%	23.5	Yes	hypochondriacus-mix_India
PI 481059	AM_00773	caudatus	IND	India	2.1X	98.7%	49.2	Yes	hypochondriacus-mix_India
PI 481061	AM_00774	caudatus	IND	India	0.9X	98.8%	62.6	Yes	hypochondriacus-mix_India
PI 481064	AM_00775	caudatus	IND	India	10.1X	98.6%	15.4	Yes	caudatus_India
PI 481066	AM_00776	caudatus	IND	India	1.3X	98.6%	51.7	Yes	hypochondriacus-mix_India
PI 481067	AM_00777	caudatus	IND	India	1.0X	98.9%	62.4	Yes	hypochondriacus-mix_India
PI 481072	AM_00778	caudatus	IND	India	8.9X	98.0%	6.8	Yes	hypochondriacus-mix_India
PI 481073	AM_00779	hybr.	IND	India	1.2X	98.5%	55.9	Yes	hybrid_India
PI 481074	AM_00780	hypochondriacus	IND	India	1.2X	98.5%	54	Yes	hypochondriacus_India
PI 481075	AM_00781	hypochondriacus	IND	India	0.9X	98.8%	59.6	Yes	hypochondriacus_India
PI 481076	AM_00782	hypochondriacus	IND	India	1.5X	98.9%	44.2	Yes	hypochondriacus_India
PI 481087	AM_00783	caudatus	IND	India	0.3X	98.4%	84.2	No	NA
PI 481090	AM_00784	hybr.	IND	India	7.8X	99.2%	11.3	Yes	hybrid_India
PI 481091	AM_00785	hypochondriacus	IND	India	0.6X	99.2%	71.6	Yes	hypochondriacus_India
PI 481092	AM_00786	hybr.	IND	India	6.1X	98.7%	19.6	Yes	hybrid_India
PI 481093	AM_00787	hypochondriacus	IND	India	0.4X	99.3%	77.4	Yes	hypochondriacus_India
PI 481098	AM_00788	caudatus	IND	India	0.3X	98.7%	87.1	No	NA
PI 481101	AM_00789	caudatus	IND	India	0.5X	98.6%	75.4	Yes	caudatus_India
PI 481106	AM_00790	hypochondriacus	IND	India	0.6X	99.2%	71.8	Yes	hypochondriacus_India
PI 481107	AM_00791	hypochondriacus	IND	India	0.4X	99.1%	77.5	Yes	hypochondriacus_India
PI 481108	AM_00792	hypochondriacus	IND	India	0.5X	98.8%	75.2	Yes	hypochondriacus_India
PI 481109	AM_00793	hypochondriacus	IND	India	0.5X	98.7%	76.1	Yes	hypochondriacus_India
PI 481110	AM_00794	hypochondriacus	IND	India	1.9X	99.0%	38	Yes	hypochondriacus_India
PI 481113	AM_00795	caudatus	IND	India	1.6X	98.8%	45.8	Yes	hypochondriacus-mix_India
PI 481116	AM_00796	caudatus	IND	India	1.3X	98.6%	49.1	Yes	hypochondriacus-mix_India

PI 481117	AM_00797	caudatus	IND	India	9.0X	98.3%	14.3	Yes	caudatus_India
PI 481124	AM_00798	caudatus	IND	India	0.4X	99.0%	78.3	Yes	hypochondriacus-mix_India
PI 481125	AM_00799	caudatus	IND	India	0.7X	98.9%	64.7	Yes	hypochondriacus-mix_India
PI 481374	AM_00800	caudatus	IND	India	1.7X	95.0%	45.9	Yes	caudatus_India
PI 615696	AM_00864	hypochondriacus	IND	India	1.6X	98.2%	46.3	Yes	hypochondriacus_India
PI 619236	AM_00865	caudatus	IND	India	2.8X	98.0%	31.8	Yes	caudatus_India
PI 636183	AM_00870	hypochondriacus	IND	India	0.8X	98.7%	65.2	Yes	hypochondriacus_India
PI 636184	AM_00871	hypochondriacus	IND	India	0.3X	98.8%	86.2	No	NA
PI 636185	AM_00872	hypochondriacus	IND	India	8.0X	98.7%	8.9	Yes	hypochondriacus_India
PI 636186	AM_00873	hypochondriacus	IND	India	0.6X	99.1%	72	Yes	hypochondriacus_India
PI 636187	AM_00874	hypochondriacus	IND	India	0.4X	98.8%	80.9	No	NA
PI 636188	AM_00875	hypochondriacus	IND	India	0.3X	98.3%	81.5	No	NA
PI 636190	AM_00876	hypochondriacus	IND	India	1.8X	96.0%	39.7	Yes	hypochondriacus_India
PI 636191	AM_00877	hypochondriacus	IND	India	1.9X	98.9%	40.7	Yes	hypochondriacus_India
PI 636192	AM_00878	hypochondriacus	IND	India	1.3X	98.3%	50.9	Yes	hypochondriacus_India
PI 636193	AM_00879	caudatus	IND	India	0.7X	98.4%	71.1	Yes	caudatus_India
PI 669855	AM_00987	caudatus	IND	India	5.6X	93.8%	26.6	Yes	caudatus_India
PI 669883	AM_00988	caudatus	IND	India	0.6X	98.7%	73.5	Yes	caudatus_India
PI 669885	AM_00989	caudatus	IND	India	0.5X	98.7%	77.5	Yes	caudatus_India
PI 669888	AM_00990	caudatus	IND	India	0.7X	98.5%	71.5	Yes	caudatus_India
PI 669889	AM_00991	caudatus	IND	India	0.1X	98.3%	92.9	No	NA
PI 669891	AM_00992	caudatus	IND	India	0.8X	98.7%	70.2	Yes	caudatus_India
PI 669895	AM_00993	caudatus	IND	India	0.9X	99.0%	62.9	Yes	hypochondriacus-mix_India
PI 669901	AM_00994	caudatus	IND	India	3.3X	98.4%	37	Yes	caudatus_India
PI 669908	AM_00995	caudatus	IND	India	4.4X	98.5%	33.5	Yes	hypochondriacus-mix_India
PI 669930	AM_00996	caudatus	IND	India	1.2X	97.9%	60.3	Yes	caudatus_India
PI 669934	AM_00997	caudatus	IND	India	0.0X	97.7%	NA	No	NA
PI 674256	AM_01009	hypochondriacus	IND	India	1.7X	97.7%	42.1	Yes	hypochondriacus_India
PI 689732	AM_01021	caudatus	IND	India	1.5X	98.4%	53.7	Yes	caudatus_India
PI 689735	AM_01023	hypochondriacus	IND	India	1.1X	98.1%	61.3	Yes	hypochondriacus_India
PI 689736	AM_01024	hypochondriacus	IND	India	0.2X	98.0%	88.5	No	NA
PI 689738	AM_01025	hypochondriacus	IND	India	0.4X	95.7%	79.5	Yes	hypochondriacus_India
PI 566897	AM_01059	cruentus	IND	India	0.8X	97.3%	67.4	Yes	cruentus_India
AMA132	AM_01094	cruentus	IND	India	0.7X	99.0%	70.7	Yes	hypochondriacus_India
AMA107	AMA107	caudatus	CHN	Native	10.4X	96.9%	1.0	No	NA
AMA125	AMA125	caudatus	PER	Native	12.4X	97.5%	9.9	Yes	caudatus_Native
AMA155	AMA155	cruentus	USA	Native	9.6X	91.1%	9.9	Yes	cruentus_Native
Ames2085	Ames2085	hypochondriacus	MEX	Native	11.1X	97.5%	3.2	Yes	hypochondriacus_Native
Ames21666	Ames21666	quitensis	ARG	Native	10.8X	96.6%	1.2	No	quitensis_Native

Ames2215	Ames2215	hypochondriacus	MEX	Native	10.0X	95.4%	3.1	No	hypochondriacus_Native
Ames5232	Ames5232	hybridus	PER	Native	8.3X	95.5%	19.9	Yes	hybridus_SA_Native
Ames5247	Ames5247	quitensis	ECU	Native	10.4X	97.3%	10.2	Yes	quitensis_Native
Ames5302	Ames5302	caudatus	PER	Native	10.0X	95.8%	10.8	Yes	caudatus_Native
Ames5334	Ames5334	quitensis	ARG	Native	11.3X	97.2%	1.5	No	quitensis_Native
Ames5335	Ames5335	hybr.	BOL	Native	8.7X	96.8%	1.0	No	NA
Ames5342	Ames5342	quitensis	PER	Native	9.4X	97.7%	9.6	Yes	quitensis_Native
Ames5457	Ames5457	hypochondriacus	MEX	Native	12.1X	98.0%	3.6	Yes	hypochondriacus_Native
Ames5552	Ames5552	cruentus	MEX	Native	9.3X	97.1%	10.3	Yes	cruentus_Native
Baernkrafft	Baernkrafft	hypochondriacus	DEU	Native	13.6X	98.0%	0.7	No	hybridus_CA_Native
PI433228	PI433228	cruentus	GTM	Native	8.0X	97.5%	10.7	Yes	cruentus_Native
PI451826	PI451826*	cruentus	GTM	Native	5.2X	93.6%	8.8	Yes	cruentus_Native
PI481949	PI481949	caudatus	PER	Native	7.3X	96.0%	2.0	No	caudatus_Native
PI481957	PI481957	caudatus	PER	Native	11.2X	97.2%	9.8	Yes	caudatus_Native
PI481960	PI481960	caudatus	PER	Native	12.7X	97.4%	9.4	Yes	caudatus_Native
PI481965	PI481965	caudatus	PER	Native	11.9X	95.1%	9.9	Yes	caudatus_Native
PI490431	PI490431	caudatus	PER	Native	11.0X	96.7%	10.3	Yes	caudatus_Native
PI490459	PI490459	caudatus	BOL	Native	12.7X	96.2%	9.8	Yes	caudatus_Native
PI490466	PI490466	quitensis	PER	Native	11.8X	97.4%	10.0	Yes	quitensis_Native
PI490489	PI490489	hybridus	PER	Native	12.1X	95.3%	18.2	Yes	hybridus_CA_Native
PI490491	PI490491	caudatus	ARG	Native	10.7X	97.1%	1.0	No	caudatus_Native
PI490511	PI490511	caudatus	PER	Native	10.8X	96.7%	1.1	No	caudatus_Native
PI490518	PI490518	caudatus	PER	Native	11.7X	96.7%	9.7	Yes	caudatus_Native
PI490561	PI490561	caudatus	PER	Native	9.3X	94.4%	11.2	Yes	caudatus_Native
PI490604	PI490604	caudatus	BOL	Native	8.0X	96.0%	11.6	Yes	caudatus_Native
PI490609	PI490609	caudatus	ECU	Native	4.3X	47.5%	17.2	Yes	caudatus_Native
PI490612	PI490612	caudatus	PER	Native	12.8X	97.5%	10.0	Yes	caudatus_Native
PI490664	PI490664*	quitensis	ECU	Native	3.8X	96.9%	0.7	No	quitensis_Native
PI490670	PI490670	quitensis	ECU	Native	10.8X	97.5%	0.9	No	quitensis_Native
PI490673	PI490673	quitensis	ECU	Native	11.1X	97.0%	10.1	Yes	quitensis_Native
PI490679	PI490679	quitensis	ECU	Native	11.1X	97.3%	0.8	No	quitensis_Native
PI490684	PI490684	quitensis	ECU	Native	11.1X	97.5%	0.9	No	quitensis_Native
PI490689	PI490689	caudaus	ECU	Native	11.0X	97.0%	1.2	No	caudatus_Native
PI490705	PI490705	quitensis	ECU	Native	11.9X	97.5%	10.1	Yes	quitensis_Native
PI490720	PI490720*	quitensis	ECU	Native	4.5X	97.3%	9.6	Yes	quitensis_Native
PI490731	PI490731	quitensis	ECU	Native	13.0X	97.8%	0.6	No	quitensis_Native
PI490739	PI490739	caudatus	ECU	Native	9.6X	97.0%	1.3	No	quitensis_Native
PI490740	PI490740	hybr.	PER	Native	10.2X	97.3%	2.2	No	NA
PI511679	PI511679	caudatus	ARG	Native	9.0X	96.7%	10.8	Yes	caudatus_Native
PI511680	PI511680	caudatus	ARG	Native	9.2X	97.2%	10.5	Yes	caudatus_Native
PI511681	PI511681	caudatus	BOL	Native	9.1X	97.7%	10.4	Yes	caudatus_Native
PI511686	PI511686	caudatus	PER	Native	10.9X	97.1%	10.4	Yes	caudatus_Native

PI511687	PI511687	caudatus	PER	Native	12.3X	96.9%	10.1	Yes	caudatus_Native
PI511690	PI511690	caudatus	PER	Native	9.4X	96.6%	11.1	Yes	caudatus_Native
PI511696	PI511696	caudatus	PER	Native	10.1X	95.8%	10.4	Yes	caudatus_Native
PI511704	PI511704	caudatus	PER	Native	12.2X	97.2%	9.8	Yes	caudatus_Native
PI511706	PI511706	caudatus	PER	Native	9.6X	96.2%	11.0	Yes	caudatus_Native
PI511712	PI511712	caudatus	ECU	Native	9.9X	96.9%	10.6	Yes	caudatus_Native
PI511713	PI511713	cruentus	PER	Native	10.9X	95.9%	9.6	Yes	cruentus_Native
PI511714	PI511714	cruentus	PER	Native	8.4X	97.4%	9.1	Yes	cruentus_Native
PI511717	PI511717	cruentus	GTM	Native	9.7X	97.0%	10.4	Yes	cruentus_Native
PI511723	PI511723	cruentus	MEX	Native	11.6X	97.9%	1.1	No	cruentus_Native
PI511724	PI511724	hybridus	MEX	Native	9.7X	95.8%	10.6	Yes	hybridus_CA_Native
PI511731	PI511731	hypochondriacus	MEX	Native	3.1X	24.6%	18.5	Yes	hypochondriacus_Native
PI511736	PI511736	quitensis	BOL	Native	9.7X	96.4%	9.4	Yes	quitensis_Native
PI511737	PI511737	quitensis	ECU	Native	12.4X	97.3%	10.0	Yes	quitensis_Native
PI511741	PI511741	quitensis	ECU	Native	13.0X	91.5%	9.6	Yes	quitensis_Native
PI511745	PI511745	quitensis	ECU	Native	10.2X	97.1%	10.7	Yes	quitensis_Native
PI511747	PI511747	quitensis	ECU	Native	9.7X	97.6%	10.4	Yes	quitensis_Native
PI511749	PI511749	quitensis	ECU	Native	11.5X	97.3%	10.1	Yes	quitensis_Native
PI511751	PI511751	quitensis	PER	Native	11.4X	97.2%	1.6	No	quitensis_Native
PI511754	PI511754	hybridus	ECU	Native	13.5X	94.6%	8.8	Yes	hybridus_SA_Native
PI511876	PI511876	cruentus	MEX	Native	11.6X	97.3%	1.3	No	cruentus_Native
PI576481	PI576481	cruentus	MEX	Native	10.2X	97.3%	9.2	Yes	cruentus_Native
PI576482	PI576482	cruentus	MEX	Native	9.9X	97.3%	10.1	Yes	cruentus_Native
PI604559	PI604559	hypochondriacus	MEX	Native	13.7X	98.3%	0.7	No	hypochondriacus_Native
PI604568	PI604568	hybridus	MEX	Native	8.3X	96.0%	16.5	Yes	hybridus_SA_Native
PI604571	PI604571	hybr.	MEX	Native	8.8X	96.6%	2.5	No	NA
PI604574	PI604574	hybridus	MEX	Native	11.6X	97.1%	9.5	Yes	hybridus_CA_Native
PI604581	PI604581	hypochondriacus	MEX	Native	11.5X	98.6%	4.5	Yes	hypochondriacus_Native
PI604582	PI604582	hybridus	MEX	Native	2.4X	23.5%	30.6	Yes	hybridus_CA_Native
PI604587	PI604587	hypochondriacus	MEX	Native	11.3X	97.1%	5.0	Yes	hypochondriacus_Native
PI604595	PI604595	hypochondriacus	MEX	Native	9.8X	97.6%	3.6	Yes	hypochondriacus_Native
PI606798	PI606798	cruentus	MEX	Native	9.5X	97.0%	10.1	Yes	cruentus_Native
PI608019	PI608019	caudatus	ECU	Native	10.5X	96.0%	10.5	Yes	caudatus_Native
PI633589	PI633589	hypochondriacus	MEX	Native	11.1X	98.1%	4.3	Yes	hypochondriacus_Native
PI636180	PI636180	hybridus	COL	Native	6.6X	89.6%	17.3	Yes	hybridus_SA_Native
PI642741	PI642741*	caudatus	BOL	Native	5.9X	94.9%	11.6	Yes	caudatus_Native
PI643036	PI643036	hypochondriacus	MEX	Native	12.0X	98.0%	3.2	Yes	hypochondriacus_Native
PI643037	PI643037	cruentus	MEX	Native	8.6X	97.7%	9.8	Yes	cruentus_Native
PI643039	PI643039	cruentus	MEX	Native	12.0X	96.6%	9.3	Yes	cruentus_Native
PI643041	PI643041	hypochondriacus	MEX	Native	12.3X	98.0%	3.5	Yes	hypochondriacus_Native
PI643042	PI643042	cruentus	MEX	Native	2.2X	80.3%	26.7	No	cruentus_Native
PI643049	PI643049	cruentus	MEX	Native	11.8X	96.5%	9.5	Yes	cruentus_Native

PI643058	PI643058	cruentus	MEX	Native	13.2X	97.1%	8.9	Yes	cruentus_Native
PI643067	PI643067	hypochondriacus	MEX	Native	11.4X	97.0%	3.7	Yes	hypochondriacus_Native
PI643070	PI643070	hypochondriacus	MEX	Native	13.2X	98.2%	2.8	Yes	hypochondriacus_Native
PI649217	PI649217	caudatus	PER	Native	9.3X	96.5%	11.0	Yes	caudatus_Native
PI649227	PI649227	caudatus	PER	Native	13.1X	95.0%	9.3	Yes	caudatus_Native
PI649228	PI649228	caudatus	PER	Native	8.9X	95.8%	11.1	Yes	caudatus_Native
PI649230	PI649230	caudatus	PER	Native	9.3X	97.0%	10.7	Yes	caudatus_Native
PI649246	PI649246	quitensis	PER	Native	10.4X	97.1%	2.0	No	quitensis_Native
PI649509	PI649509	cruentus	MEX	Native	9.7X	97.3%	10.1	Yes	cruentus_Native
PI649514	PI649514	cruentus	MEX	Native	7.0X	62.8%	11.6	Yes	cruentus_Native
PI649524	PI649524	cruentus	MEX	Native	10.4X	96.4%	9.7	Yes	cruentus_Native
PI649529	PI649529	hypochondriacus	MEX	Native	9.4X	97.3%	3.2	Yes	hypochondriacus_Native
PI649537	PI649537	hypochondriacus	MEX	Native	12.0X	97.9%	3.6	Yes	hypochondriacus_Native
PI649559	PI649559	hypochondriacus	MEX	Native	10.8X	97.1%	3.8	Yes	hypochondriacus_Native
PI649565	PI649565	hypochondriacus	MEX	Native	10.6X	97.9%	1.0	No	hypochondriacus_Native
PI649575	PI649575	hypochondriacus	MEX	Native	10.5X	97.9%	3.5	Yes	hypochondriacus_Native
PI649587	PI649587	hypochondriacus	MEX	Native	9.5X	96.7%	2.5	No	hypochondriacus_Native
PI649595	PI649595	hypochondriacus	MEX	Native	12.2X	97.8%	0.8	No	hypochondriacus_Native
PI649602	PI649602	hypochondriacus	MEX	Native	11.8X	97.8%	2.5	Yes	hypochondriacus_Native
PI649607	PI649607	hypochondriacus	MEX	Native	11.5X	97.9%	3.7	Yes	hypochondriacus_Native
PI649609	PI649609	cruentus	MEX	Native	9.6X	93.5%	10.0	Yes	cruentus_Native
PI649623	PI649623	hypochondriacus	MEX	Native	10.9X	97.4%	2.9	Yes	hypochondriacus_Native
PI652422	PI652422	quitensis	BRA	Native	13.6X	97.3%	1.1	No	quitensis_Native
PI652426	PI652426	quitensis	BRA	Native	10.2X	96.2%	1.7	No	quitensis_Native
PI652428	PI652428	quitensis	BRA	Native	11.9X	97.1%	1.4	No	quitensis_Native
PI652432	PI652432	hypochondriacus	BRA	Native	10.7X	97.3%	2.5	No	hypochondriacus_Native
PI658728	PI658728	cruentus	MEX	Native	9.5X	97.0%	9.8	Yes	cruentus_Native
PI667156	PI667156	quitensis	ECU	Native	10.2X	97.3%	1.4	No	quitensis_Native
PI667158	PI667158	hybridus	GTM	Native	10.1X	97.0%	7.0	Yes	hybridus_CA_Native
PI667160	PI667160	cruentus	GTM	Native	9.0X	96.4%	10.3	Yes	cruentus_Native
PI667165	PI667165	cruentus	BRA	Native	9.7X	97.0%	10.2	Yes	cruentus_Native

* These accessions were sub-sampled to map the coverage of the samples

Table S2 List of genes annotated in 10kb up-downstream region of assocaited GWAS SNP.

Chromosome	Start	End	Name	Description
1	54290	74290	AHp000004	lysine-specific demethylase
4	2539899	2559899	AHp006724	L-galactono-1,4-lactone dehydrogenase
5	18969424	18989424	AHp009270	malic enzyme
9	20373084	20393084	AHp015126	isoform X1
10	18379508	18399508	AHp016221	Signal peptidase complex subunit
10	18379508	18399508	AHp016222	DOMON domain-containing protein
11	2043844	2063844	AHp016706	Belongs to the protein kinase superfamily. Ser Thr protein kinase family
11	22024217	22044217	AHp017947	Boron transporter
11	22024217	22044217	AHp017948	Catalyzes the phosphorylation of pantothenate the first step in CoA biosynthesis. May play a role in the physiological regulation of the intracellular CoA concentration
12	16842318	16862318	AHp019109	Domain of unknown function (DUF4336)
12	16842318	16862318	AHp019110	ribulose-phosphate 3-epimerase
13	5600536	5620536	AHp019501	disulfide-isomerase
15	9943938	9963938	AHp022473	histone H2B
15	9943938	9963938	AHp022474	histone H2B

Table S3 Genome-wide average of different population genetics statistics for each population calculated using angsd.

Population	Region	Pi	Tajimas' D	Watterson Theta
A. hybridus	Wild	0.00859	0.01600	0.00856
A. quitensis		0.00149	-0.59352	0.00159
A. caudatus	Native	0.00276	-0.09276	0.00275
A. cruentus		0.00412	-0.43050	0.00454
A. hypochondriacus		0.00339	-0.62971	0.00391
A. caudatus	India	0.00405	-2.69082	0.01564
A. cruentus		0.00398	-1.42465	0.00536
A. hypochondriacus		0.00377	-2.93702	0.02763
Hypochondriacus_mix		0.00333	-2.97796	0.03519
Hybrid		0.00328	-2.89583	0.01617

Table S4 95% confidence interval along mean nucleotide diversity estimated 50 times by sub-sampling 10 individuals from each population at a time using angsd.

Species		Pi		Tajimas D		Wattersons Theta	
	Region	95% CI along mean Pi		95% CI along mean D		95% CI along mean theta	
		LowerBound	UpperBound	LowerBound	UpperBound	LowerBound	UpperBound
A. hybridus	Wild	0.008589	0.008593	0.01574	0.016089	0.008558	0.008563
A. quitensis		0.001854	0.00414	-0.803274	-0.055105	0.002083	0.005073
A. caudatus	Native	0.002349	0.002955	0.00375	0.620444	0.002028	0.002778
A. cruentus		0.002241	0.005253	-0.630545	0.541257	0.002045	0.004846
A. hypochondriacus		0.002584	0.005005	-0.781551	0.32704	0.002374	0.004926
A. caudatus	India	0.003213	0.00473	-2.4797	-2.09447	0.007762	0.010296
A. cruentus		0.003984	0.003985	-1.43373	-1.43258	0.005363	0.005365
A. hypochondriacus		0.003243	0.004436	-2.56322	-2.41329	0.008364	0.010645
Hypochondriacus-mix		0.002665	0.003784	-2.58158	-2.5433	0.006844	0.00993
Hybrid		0.003125	0.003513	-2.57504	-2.55856	0.008078	0.00918

Table S5 Parameter estimates of best-fit demographic model for the two species with 95% confidence interval.

**Native Bottleneck- introduced expansion- continuous gene flow**			
A. hypochondriacus			
**Parameters**	**PointEstimates**	**LowerBound-05**	**UpperBound-95**
**ANCSIZE**	14693	10470	130783
**NATIVE**	1615042	10153	8543408
**NPOPOUT**	277553	120469	973019
**NBOT**	1355	1015	764414
**INDIA**	38513	27919	341274
**TDIV**	1240	908	9844
**TBOT**	15	11	8627
**R1**	-0.00562649	-0.007668	-0.000707
**MIG12**	0.0011074	0.000142	0.001467
**MIG21**	0.000189159	2.3E-05	0.000265
**TDUR**	653	132	905
**TENDBOT**	667	382	9008
**MaxEstLhood**	-6009456.677	-108604.808	-105911.954
**MaxObsLhood**	-5936545.021	-108356.491	-105788.505

**Native Bottleneck- introduced expansion- Introgression with unknown population**			
A. caudatus			
**Parameters**	**Point Estimates**	**LowerBound-05**	**UpperBound-95**
**ANCSIZE**	649899	384442	1040340
**NATIVE**	3069064	32756	9509859
**NPOPOUT**	243883	111614	910672
**NBOT**	1108	1012	1979
**INDIA**	9868060	5645064	12346802
**TDIV**	2666	1260	6051
**TBOT**	383	137	648
**R1**	-0.00416919	-0.009469	-0.001713
**MIG13**	0.0020243	1.9E-05	0.003223
**TDUR**	181	158	320
**TENDBOT**	565	333	934
**MaxEstLhood**	-5030246.35	-987420.829	-980605.148
**MaxObsLhood**	-4970605.226	-987286.588	-980480.136

Table S6 List of genes within 10kb up-downstream region of the putative selective sweeps identified using XP-CLR.

Table S7 Stepwise forward variable selection procedure using ordiR2step. The stopping criteria used were - variable significance of $p < 0.01$ using 1000 permutations, and the adjusted R^2 of the global model.

Variable	R square	Cumulative R square	F value	P value
A. caudatus				
Bio3	0.108	0.108	6.1092	0.002 **
Bio9	0.009	0.117	1.423	0.018 *
A. hypochondriacus				
Bio2	0.094	0.094	4.3075	0.002 **
Bio7	0.014	0.109	1.5251	0.004 **

Table S8 Full and partial Redundancy analysis (RDA) to partition variances into genetic, climatic and geographic components.

Partial RDA models	Inertia	R 2	p (>F)	Proportion of explainable variance	Proportion of total variance
A. caudatus					
Full model: F ∼clim. + geog. + struct.	4076.4	0.38	0.001 ***	1	0.31
Pure climate: F ∼clim. ∣ (geog. + struct.)	423.8	0.039	0.24	0.1	0.032
Pure structure: F ∼struct. ∣ (clim. + geog.)	1423.7	0.13	0.001 ***	0.34	0.108
Pure geography: F ∼geog. ∣ (clim. + struct.)	617.2	0.057	0.244	0.15	0.046
Total unexplained	6635.4				0.5
Total inertia	13176.5				1
A. hypochondriacus					
Full model: F ∼clim. + geog. + struct.	1252.6	0.413	0.001 ***	1	0.32
Pure climate: F ∼clim. ∣ (geog. + struct.)	170.35	0.056	0.118	0.136	0.044
Pure structure: F ∼struct. ∣ (clim. + geog.)	474.35	0.157	0.001 ***	0.379	0.121
Pure geography: F ∼geog. ∣ (clim. + struct.)	237.78	0.079	0.223	0.19	0.061
Total unexplained	1773.4				0.454
Total inertia	3908.48				1

Table S9 List of annotated genes in the 20kb up-downstream regions of significantly climate associated loci identified using RDA
